# Sleep magnetoencephalography enhances detection and source imaging of seizures and fast oscillations in focal cortical dysplasia

**DOI:** 10.1002/epi.70191

**Published:** 2026-03-10

**Authors:** Marcel Heers, Jawata Afnan, Christoph Braun, Christophe Grova, Dirk‐Matthias Altenmüller, Bernhard J. Steinhoff, Matthias Dümpelmann, Theo Demerath, Horst Urbach, Silke Ethofer, Markus Siegel, Andreas Schulze‐Bonhage, Holger Lerche, Yiwen Li Hegner

**Affiliations:** ^1^ Epilepsy Center Medical Center–University of Freiburg, Faculty of Medicine, University of Freiburg Freiburg Germany; ^2^ Multimodal Functional Imaging Lab Montreal Neurological Institute, McGill University Montreal Quebec Canada; ^3^ Magnetoencephalography Center University of Tübingen Tübingen Germany; ^4^ Department of Neural Dynamics and Magnetoencephalography Hertie Institute for Clinical Brain Research, University of Tübingen Tübingen Germany; ^5^ Center for Integrative Neuroscience University of Tübingen Tübingen Germany; ^6^ Centre De Recherches Mathématiques Montreal Quebec Canada; ^7^ Multimodal Functional Imaging Lab, Department of Physics and PERFORM Center Concordia University Montreal Quebec Canada; ^8^ Epilepsy Center Kork Kehl‐Kork Germany; ^9^ Department of Microsystems Engineering–IMTEK University of Freiburg Freiburg Germany; ^10^ BrainLinks–BrainTools Center University of Freiburg Freiburg Germany; ^11^ Department of Neuroradiology Medical Center–University of Freiburg, Faculty of Medicine, University of Freiburg Freiburg Germany; ^12^ Department of Neurosurgery University Hospital Tübingen Tübingen Germany; ^13^ Center for Bionic Intelligence Tübingen Stuttgart Tübingen Germany; ^14^ German Center for Mental Health Tübingen Germany; ^15^ Department of Neurology and Epileptology Hertie Institute for Clinical Brain Research, University of Tübingen Tübingen Germany

**Keywords:** focal cortical dysplasia, focal epilepsy, magnetic source imaging, seizures, sleep

## Abstract

**Objective:**

Focal cortical dysplasia (FCD) causes drug‐resistant epilepsy requiring presurgical evaluation. Invasive electroencephalographic (EEG) studies demonstrate that sleep modulates epileptic activity, including interictal epileptiform discharges (IEDs), fast oscillations (FOs) in the beta (14–40 Hz) and gamma (40–80 Hz) frequency bands, and seizures. This study aimed to quantify sleep‐associated changes in IEDs, FOs, and seizures in FCD patients using noninvasive magnetoencephalography (MEG).

**Methods:**

Nineteen patients with FCD were prospectively recruited and underwent simultaneous MEG/EEG recordings lasting 89 ± 19 min during daytime sleep. Sleep stages were classified from the EEG. Beamformer source signals were computed from the MEG signal to enhance sensitivity for visual detection of IEDs, FOs in the beta and gamma frequency bands, and seizures. Magnetic source imaging (MSI) was performed using the Maximum Entropy on the Mean (MEM) method, which is particularly sensitive to the spatial extent of sources, enabling accurate localization of epileptic activity.

**Results:**

N1 sleep was reached in 17 of 19 patients and N2 sleep in 14 of 19 patients. Compared to wakefulness, sleep recordings showed significantly higher rates of FOs and seizures (both *p* < .05), whereas IED rates showed nonsignificant trends. Ten patients demonstrated FOs or seizures, and 12 showed IEDs. MSI of IEDs demonstrated consistent accuracy across vigilance states, with median Euclidean distances of 12.74 mm (interquartile range [IQR] = 22.74) in wake and 8.34 mm (IQR = 27.58) in sleep, and no systematic amplitude or spatial extent changes. Wavelet‐MEM enabled frequency‐specific source imaging, with FOs and seizures localizing concordantly to FCD lesions in five of seven and seven of eight patients, respectively.

**Significance:**

Daytime sleep MEG recordings are clinically feasible and significantly enhance the detection of seizures (37% of patients) and FOs compared to wakefulness. Sleep protocols enable noninvasive capture of ictal patterns—the gold standard for epileptogenic zone localization—alongside increased FO rates. These findings support incorporating sleep into standard MEG protocols for presurgical epilepsy evaluation.

**Key points**

- Sleep MEG captures seizures in 37% of FCD patients, the gold standard for epileptogenic zone localization.
- Beta and gamma fast oscillations increase significantly during sleep, providing additive localization beyond IEDs.
- Ninety‐minute daytime recordings with sleep yield substantially more diagnostic information than wake‐only protocols.
- Source imaging concordance across multiple biomarkers during sleep enhances characterization of the epileptogenic zone.

## 1 INTRODUCTION

Malformations of cortical development frequently cause drug‐resistant focal epilepsy.^1^, ^2^ Focal cortical dysplasia (FCD) type II, characterized by dysmorphic neurons with or without balloon cells, represents the most common subtype.^2^ Additional malformations include FCD type I, characterized by abnormal cortical architecture, and mild malformation of cortical development (mMCD).^3^

Presurgical evaluation of FCD requires accurate localization of epileptogenic tissue. Although magnetic resonance imaging (MRI) provides structural identification,^4^, ^5^, ^6^ electroclinical correlation is essential.^7^, ^8^, ^9^ Complete resection of well‐defined type II FCDs predicts favorable surgical outcomes^10^; subtle lesions typically require invasive EEG to delineate the seizure onset zone (SOZ).^11^, ^12^ Magnetoencephalography (MEG) provides complementary noninvasive localization, with concordance between MEG sources and FCD lesions predicting favorable postsurgical outcomes.^9^, ^13^ Optimizing MEG protocols is essential to maximize diagnostic contribution within multimodal presurgical workflows.

Invasive electroencephalographic (EEG) studies in FCD patients reveal state‐dependent patterns of epileptic activity. Awake recordings typically show repetitive interictal epileptiform discharges (IEDs), whereas sleep recordings demonstrate increased fast oscillations (FOs) in beta and gamma frequency bands.^14^, ^15^, ^16^, ^17^ Sleep also increases seizure occurrence in FCD patients.^18^, ^19^ These sleep‐related changes reflect pathophysiological interactions between brain states and epileptic activity that remain incompletely understood. Still, they may help identify factors that increase seizure risk in FCD patients.^18^, ^20^ Notably, seizures provide the gold standard localization of epileptogenic tissue, making their detection a priority for presurgical evaluation.

MEG combined with simultaneous EEG provides advantages for tracking sleep‐related changes in FCD patients. MEG offers noninvasive whole‐head coverage with high sensitivity for superficial cortical sources, ideal for detecting activity from FCDs, which typically occur in neocortical regions.^21^, ^22^ Its reference‐free sensors and silent operation facilitate sleep recordings while maintaining spatial precision.^23^, ^24^ Sleep significantly enhances the detection of FOs in noninvasive recordings. Pathological FOs are detected more frequently during sleep than wakefulness.^25^, ^26^, ^27^ Although MEG shows lower FO detection rates compared to scalp EEG, MEG FOs demonstrate higher specificity and localization accuracy for the epileptogenic zone.^26^, ^27^ These complementary properties suggest that sleep MEG protocols could optimize detection of pathological FOs while maintaining the spatial precision needed for presurgical evaluation. Therefore, incorporating sleep into MEG recordings may capture clinically relevant biomarkers that standard wake‐only protocols frequently miss.

Based on invasive EEG observations, we hypothesized that sleep‐related changes in epileptic activity could be demonstrated noninvasively with MEG in FCD patients. Specifically, we hypothesized that rates of IEDs, FOs, and seizures increase during sleep. Beyond detection rates, we aimed to determine whether sleep recordings enable robust source imaging of multiple types of epileptic activity and whether spatial concordance between biomarkers provides validation of the epileptogenic zone.

We recorded simultaneous MEG/EEG during daytime sleep in FCD patients to maximize diagnostic yield. Beamformer‐based virtual sensors positioned within lesional tissue^27^ enhanced detection sensitivity for IEDs, FOs, and seizures, which were compared against homologous contralateral control regions.

## 2 MATERIALS AND METHODS

### 2.1 Patient characteristics and ethics

Between July 2020 and September 2025, a total of 29 patients were screened. Two patients had suspected deep lesions, and subsequent invasive stereo‐EEG (sEEG) revealed multifocal bihemispheric epilepsy. MEG could not be performed in two other patients due to strong artifacts caused by a dental prosthesis in one patient and extensive tattoos in the other. Five patients could not be motivated to participate in the study due to the requirement to travel several hours to the MEG laboratory (Figure S1).

We prospectively recruited 19 epilepsy patients with FCDs from the epilepsy surgery program of the University Epilepsy Centers in Freiburg and Tübingen, and the Epilepsy Center Kork, Kehl‐Kork, Germany. This human study was approved by both of the local ethics committees in Freiburg (approval numbers Freiburg/Kork: 21‐1666/23‐1557‐S1) and Tübingen (approval number: 344/2021) and was conducted in accordance with the Declaration of Helsinki. All patients gave written informed consent before participating in this study. The study was registered at the German Clinical Trials Register (DRKS00031233). The cohort included patients with malformation subtypes (potentially FCD types I and II, mMCD). Throughout this article, we use the term FCD to refer to all of them.

### 2.2 Simultaneous MEG/EEG recordings

All patients underwent approximately 90‐min (with a minimum of 60 min) eyes‐closed, resting‐state MEG and EEG (MEG/EEG) recordings in a supine position during daytime. Patients were asked to get up early in the morning to be able to sleep during the recordings, but we did not follow a more extended sleep deprivation protocol. Patients were not administered pharmacological sleep aids. Although pharmacological approaches such as clonidine (known to enhance IEDs in MEG)^28^ and etomidate (known to activate FOs in the ripple band in intracranial EEG)^29^ have been used in prior epilepsy studies, we employed a nonpharmacological approach relying on natural sleep induction through early morning awakening and extended recording duration to avoid confounding effects on epileptic activity. In seven patients, an additional 10‐min interleaved eyes‐open/eyes‐closed protocol (2.5 min each) was acquired to balance wake and sleep data, because initial recordings revealed disproportionately long sleep periods in some patients. MEG data were acquired with a 270‐channel whole‐head gradiometer system (five channels had defects in 275 channels, CTF) with a sampling rate of 1172 Hz in a magnetically shielded room (VSM MedTech). Simultaneously, EEG was recorded from 32 scalp electrodes mounted in an EasyCap (BrainProducts) according to the international 10–20 system (CPz as reference, ground at left clavicle) and two electrocardiographic electrodes on the right clavicle and left abdomen. Before cap placement, the head shape of each patient (nasion, bilateral preauricular points, and ~400 scalp points) was digitized using a Polhemus FastTrack system; after EEG cap mounting, electrode positions were additionally digitized. Continuous head position was monitored throughout acquisition via CTF head‐localization coils.

### 2.3 MEG/EEG sensor‐level preprocessing

Raw MEG/EEG data were visually inspected, and artifact‐contaminated segments were marked. Data were analyzed using custom MATLAB algorithms (MathWorks) and Fieldtrip.^30^ MEG channels were high‐pass filtered at .1 Hz (first‐order Butterworth) and EEG channels at 1 Hz (fourth‐order Butterworth) to account for different noise characteristics. Line noise (50 Hz and harmonics) was attenuated via adaptive notch filtering. Independent component analysis identified ocular and cardiac artifacts by topography and time course; these components were removed. Data were then low‐pass filtered at 160 Hz and downsampled to 640 Hz for subsequent analysis.

### 2.4 MRI segmentation and coregistration

Every patient received a high‐resolution (1 mm isotropic) T1‐weighted whole‐head structural scan (three dimensional [3D] magnetization‐prepared rapid acquisition gradient echo). Individual MRI scans were segmented using FreeSurfer (http://surfer.nmr.mgh.harvard.edu/), with cortical meshes standardized to 642 vertices per hemisphere via SUMA (https://afni.nimh.nih.gov/Suma). Patients from the Epilepsy Center Freiburg additionally underwent MRI morphometry using MAP18 and the MELD algorithm.^6^, ^31^ MRI‐to‐MEG/EEG coregistration employed fiducial alignment (nasion, preauricular points), fine‐tuned by matching digitized head shape points in FieldTrip and Brainstorm.^32^

### 2.5 Source‐level signal with linear constrained minimum variance beamformer

To enhance sensitivity for FOs, IEDs, and seizure patterns, continuous source traces were generated using linear constrained minimum variance (LCMV) beamforming.^33^ A single‐shell individual boundary element brain model was created in FieldTrip with leadfields computed using rank reduction, normalization, and source points at the gray–white matter boundary.

LCMV beamforming (λ = 5%, NAI normalization, fixed orientation) was applied to artifact‐free data to derive spatial filters, which were then applied to continuous data (including artifacts) to extract source time courses at each cortical vertex. Beamformer signals were used for subsequent marking of IEDs, FOs, and seizure patterns.

### 2.6 Region of interest definition, marking of IEDs, FOs, and seizures, and state comparison

An experienced epileptologist (M.H.) visually identified the FCD lesion center on the SUMA mesh and its contralateral homologue, in agreement with clinical neuroradiological reports. The FCD lesion extent was visually marked on segmentations of the cortical surface based on MRI criteria, considering coregistered 3D fluid‐attenuated inversion recovery and T1 MRI sequences of the individual patients. In the two patients with mMCD (ID1, ID7), the SOZ identified on sEEG—encompassing all recorded contacts and the cortical tissue between them—was used as ground truth after coregistration of postimplantation MRI and visual marking of sEEG electrode positions in Brainstorm software, typically extending beyond the MRI‐visible lesional zone. The visually delineated lesion extent served as ground truth for source imaging accuracy, based on established intrinsic epileptogenicity^34^ and correlation between complete resection and seizure freedom.^10^

**FIGURE 1:**
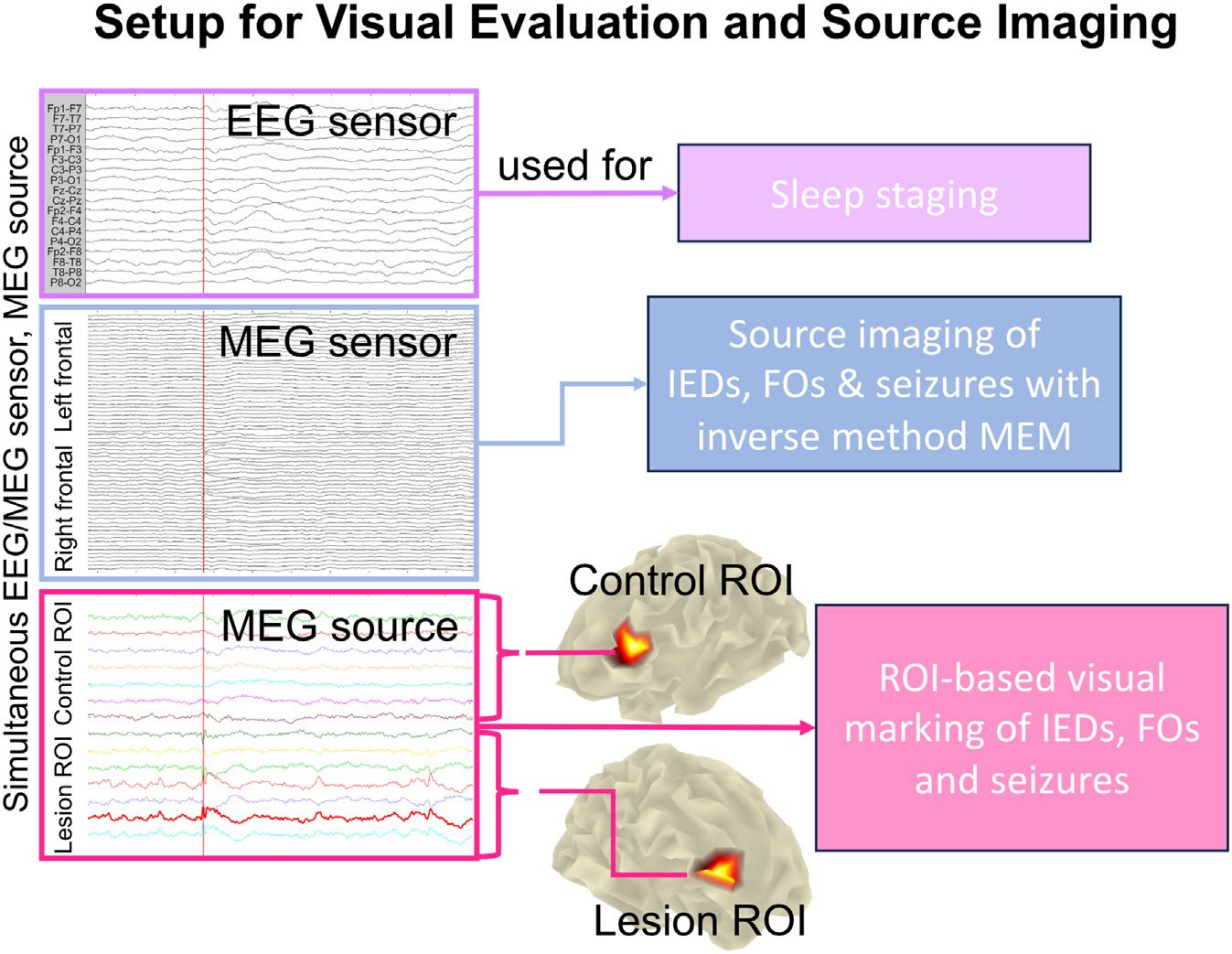
Setup for simultaneous magnetoencephalographic (MEG)/electroencephalographic (EEG) recording and source imaging analysis. The experimental workflow proceeds from top to bottom: (top) EEG sensor data used for sleep staging, (middle) MEG sensor data used for source imaging, and (bottom) source‐level traces extracted from lesional and contralateral control regions of interest (ROIs). Interictal epileptiform discharges (IEDs), fast oscillations (FOs), and seizures were visually identified in the source‐level traces within a lesional ROI and a homologous contralateral control ROI. Source imaging was performed using the Maximum Entropy on the Mean (MEM) method applied to MEG data. The brain images show the spatial locations of the lesional ROI (bottom brain) and control ROI (top brain) used for source‐level analysis.

Datasets comprising 270 MEG channels, 31 EEG channels (six in two patients with large head size), and ~14 virtual channels in the two source‐level regions of interest (ROIs; seven lesional, seven contralateral virtual channels) were exported to Brainstorm software.^32^ Virtual channel traces were inspected alongside sensor‐level MEG/EEG to differentiate physiological sleep‐related changes from epileptic activity (IEDs, FOs, seizures) within the focus. Sleep stages were classified in 30‐s epochs per the American Association of Sleep Medicine criteria.^35^ FOs were defined as ≥4 consecutive oscillatory cycles exceeding background activity in beta (14–40 Hz) or gamma (40–80 Hz) bands.^26^, ^36^ Two expert reviewers (M.H., Y.L.H.) independently classified IEDs, FOs, seizures, and sleep stages. Initial disagreements were jointly rereviewed until consensus, ensuring high classification confidence (Figure 1).

### 2.7 Distributed magnetic source imaging of averaged IEDs, FOs, and seizures

#### 2.7.1 Inverse method and forward model

For magnetic source imaging (MSI) of IEDs, FOs, and seizures, we employed the Maximum Entropy on the Mean (MEM) inverse method. The MEM method was selected based on rigorous testing demonstrating its superior sensitivity to the spatial extent of sources compared to other standard inverse methods.^37^, ^38^, ^39^ For the forward model, a single‐compartment head model was created using OpenMEEG^40^ within the Brainstorm software environment.

#### 2.7.2 Data selection and preprocessing

For MSI analysis, raw MEG data were high‐pass filtered at 2 Hz. Equal numbers of randomly selected IEDs were averaged separately for wake and sleep segments in each patient. Different sleep stages (N1–N3) were not subdivided, as further differentiation would reduce event counts below thresholds for reliable analysis. Epoch windows comprised 1 s for IEDs and FOs (.4 s pre‐peak/onset, .6 s post) and 6 s for seizures (starting 4 s preonset). For IED analysis, a consistent 2‐s wake‐state baseline was applied to both wake and sleep conditions to prevent noise‐related variations in source spatial extent comparisons.^41^ Similarly, for FO and seizure analyses, a 2‐s wake baseline was used.

#### 2.7.3 MSI implementation

MSI was performed using coherent MEM (cMEM)^38^, ^42^ for spike localization and wavelet‐MEM (wMEM)^26^, ^43^, ^44^ for FO and seizures. MEM provides accurate localization of the generators together with their spatial extent, as demonstrated by the standard variant of MEM, cMEM,^38^, ^42^ as well as the wavelet‐based extension, wMEM.^26^, ^43^, ^44^

We computed source localization metrics at the peak of the averaged IED to maximize signal‐to‐noise ratio. The −50 ms to +50 ms window was used for spatial clustering in cMEM, with metrics (Euclidean distance to the lesion [Dmin], spatial dispersion [SD], area under the receiver operating characteristic curve [AUC]) evaluated at the peak.^39^, ^45^ Analysis of the rising phase,^42^, ^46^ which addresses IED propagation, was beyond the scope of this study.^26^, ^43^, ^44^

For FOs and seizures, wMEM applies discrete wavelet transformation (Daubechies wavelets) to characterize oscillatory patterns. Data were resampled to appropriate sampling frequencies (e.g., 256 Hz for 16–32 Hz band analysis). In FCD patients, seizure onsets often show rhythmic beta–gamma patterns (14–80 Hz).^15^, ^16^ Patient‐specific frequency bands for wMEM source localization were determined individually based on the dominant spectral content of each FO's or seizure's initial period, identified through time–frequency and spectral analysis, enabling frequency‐specific source imaging.^44^ Reconstructed FO signals were averaged between 0 and .6 s. For seizures, principal component analysis was applied to the first 2 s of source‐reconstructed data.^26^ Although wMEM resolves sources at 100 ms temporal scales, we used standardized 2‐s intervals to ensure consistent methodology.

### 2.8 Quantification of differences between wake and sleep

To systematically evaluate the effect of sleep on epileptic activity, we quantified differences between wake and sleep states using several metrics. Event rates (IEDs, FOs, and seizures per minute) were compared between wake and sleep using the Wilcoxon signed‐rank test with false discovery rate (FDR) correction to assess whether sleep increased detection of epileptic activity. Amplitude peaks of the same counts of averaged IEDs and slow waves per IED type were compared between states using equal numbers of randomly averaged events, with slow wave maxima identified 20–200 ms post‐IED peak.

Three metrics evaluated MSI performance of IEDs against visually marked ground truth of lesion extent based on MRI criteria (for details, see Section 2.6): SD quantified the spatial spread of the localization around the FCD lesion, Dmin measured the straight‐line distance between source maximum and the closest vertex belonging to the FCD lesion, and AUC assessed overlap between MSI findings and lesion location across source strength thresholds.^47^, ^48^, ^49^ For FOs and seizures, sublobar concordance was additionally evaluated by determining whether the source maximum localized to the same sublobar region as the FCD lesion, following previously established methods.^37^, ^45^ For all biomarkers (IEDs, FOs, and seizures), results were classified as concordant when showing sublobar concordance with Euclidean distance ≤ 25 mm to the lesion border, and discordant when showing sublobar mismatch or Euclidean distance > 25 mm to the lesion border.

## 3 RESULTS

### 3.1 Patient characteristics

This study included 19 patients with FCD. The median age was 34 years (range = 19–67 years), and the median age of epilepsy onset was 14 years (range = 2–15 years). Fifteen patients had frontal lobe epilepsy, whereas four had FCDs outside the frontal lobe. One patient had two distinct FCDs: one in the temporal–occipital region and one in the postcentral region. Based on video‐EEG monitoring, 11 of 19 patients (58%) had sleep‐related epilepsy, defined as more than two‐thirds of seizures occurring during sleep.^18^

### 3.2 Sleep duration

Seventeen of the 19 patients were able to fall asleep. Two patients reached only N1 stage, 14 patients progressed to N2 stage, and one patient reached N3 stage. The mean sleep durations were 22 ± 11.9 min for N1 (*n* = 17) and 25 ± 18.7 min for N2 (*n* = 14), and the mean wake duration was 33 ± 20 min (*n* = 19), with considerable variability between patients. The mean total recording duration was 89 ± 19 min (*n* = 19; see Table S1 for details).

### 3.3 Interictal epileptiform discharges

**FIGURE 2:**
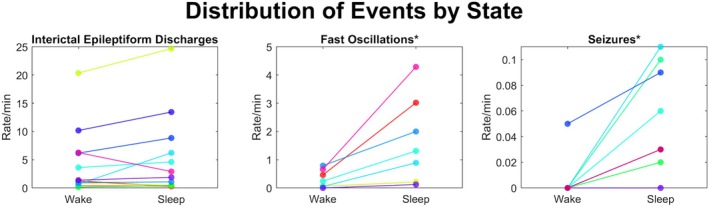
Distribution of events by sleep–wake state across patients. The figure shows significantly higher rates of fast oscillations and seizures during sleep than during the wake state (both *p* = .02, false discovery rate [FDR]‐corrected Wilcoxon signed‐rank test, *α* = .05). Interictal epileptiform discharges did not differ significantly between states (*p* = .18). Each colored line represents an individual patient, with connected points showing the event rate per minute during wake and sleep states. *Statistical significance using the Wilcoxon signed‐rank test (FDR‐corrected, *α* = .05).

Of the 19 included patients, 12 patients (63%) showed IEDs, representing 71% of patients who achieved sleep during recordings. IEDs were detected within the lesion ROI but not within the contralateral ROI. IED rates showed a moderate increase during sleep (5.43 ± 7.31 per minute) compared to wake (4.35 ± 5.93 per minute), although this difference was not statistically significant (*p* = .18; Figure 2).

### 3.4 Amplitude and source imaging characteristics

**FIGURE 3:**
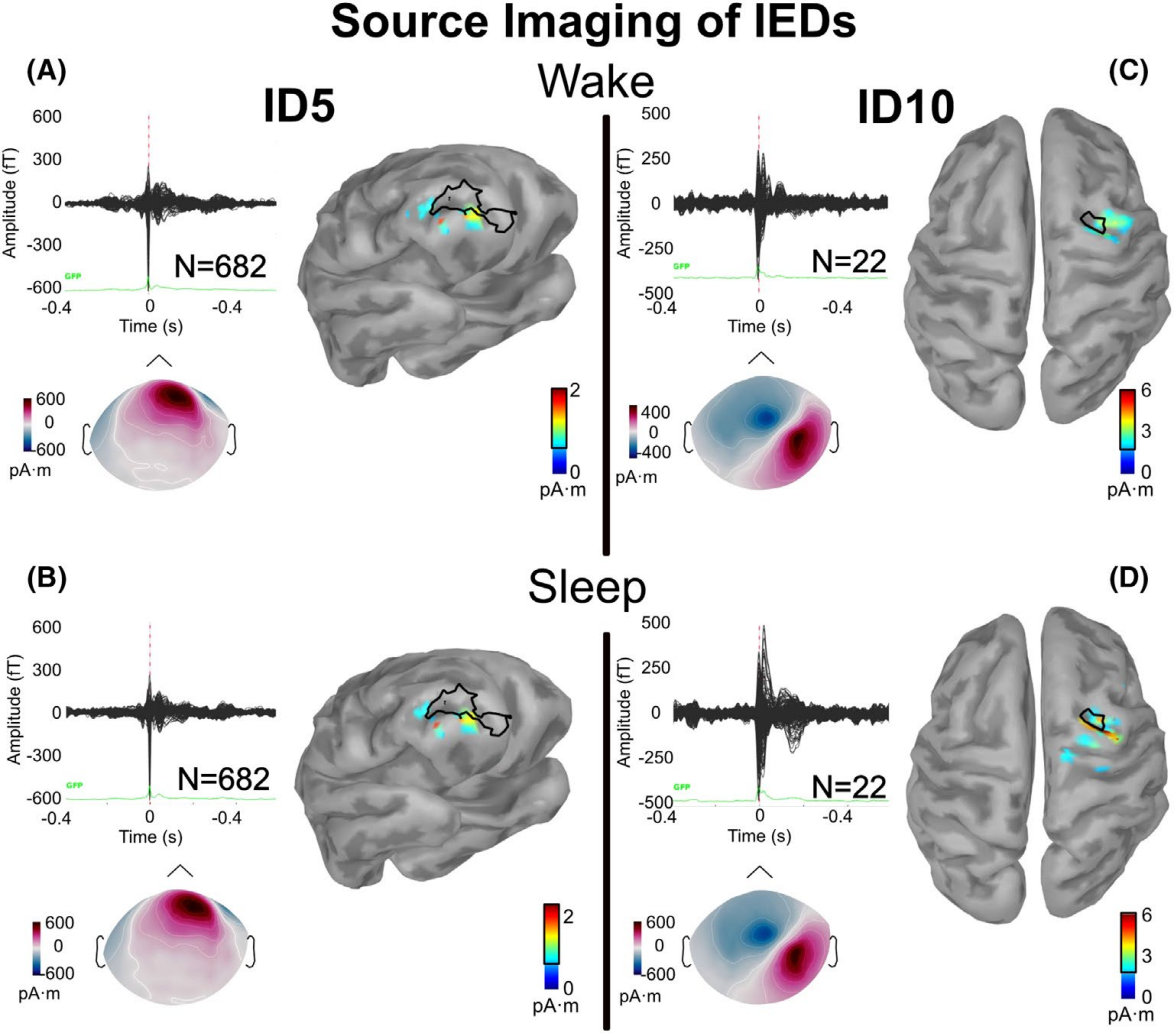
Magnetic source imaging of interictal epileptiform discharges (IEDs) analyzed with coherent Maximum Entropy on the Mean, comparing wake and sleep states in two patients. (A, B) Patient ID5 during wake (A) and sleep (B) with *n* = 682 averaged IEDs per condition. (C, D) Patient ID10 during wake (C) and sleep (D) with *n* = 22 averaged IEDs per condition. Each panel shows (left) butterfly plot of magnetoencephalography (MEG) sensor waveforms with the amplitude topography map below and (right) source imaging results projected onto cortical surface models. Black lines on cortical models outline focal cortical dysplasia (FCD) lesion borders visible on magnetic resonance imaging. In both datasets, MEG source maxima localized to the sulcal wall at the border of the FCD in direct contact with the lesion border (Euclidean distance to the lesion = 0); FCDs are typically confined to the bottom of the sulcus. Source maps display only the upper 70% of the source maximum to enhance contrast. Note increased IED and slow wave amplitudes during sleep in ID10, with slightly increased source extent. Increased spike and slow wave amplitudes were not systematically observed in our patient group. Amplitude scales differ between patients to optimize visualization.

MSI of IEDs with cMEM demonstrated good accuracy in both vigilance states, with median localization error (Dmin) to FCD lesions of 12.74 mm (interquartile range [IQR] = 22.74) during wake and 8.34 mm (IQR = 27.58) during sleep. All parameters of localization accuracy, including SD and AUC, showed no substantial differences between wake and sleep states (all *p* > .05; Table S2). The primary determinant of localization metrics was IED count rather than vigilance state, with trends toward negative correlations observed between IED count and both SD and Dmin in both conditions (Figure S2). Similarly, comparison of averaged IEDs created from equal numbers of events revealed no systematic amplitude changes in IED peaks or slow wave maxima across the cohort between states (Table S1), although in individual patients with high IED counts enabling robust averaging, increased peak amplitudes of both IEDs and slow waves were observed during sleep (Figure 3, Patient ID10).

### 3.5 FOs and seizures during sleep

#### 3.5.1 Detection rates and state‐dependent changes

FOs were detected in seven of 12 patients (58%) who showed IEDs. FO rates increased substantially from .32 ± .32 per minute during wake to 1.69 ± 1.53 per minute during sleep (*p* = .02, FDR‐corrected; *r* = .63), with all patients showing higher rates during sleep (Figure 2). Seizures occurred in seven patients during sleep recordings. Seizure rates similarly increased significantly from .01 ± .02 per minute during wake to .04 ± .04 per minute during sleep (*p* = .02, FDR‐corrected; *r* = .63), with all seven patients with seizures showing increased seizure activity during sleep (Figure 2). Notably, all patients with FOs also demonstrated IEDs, whereas three of seven patients with seizures (ID8, ID9, and ID19) lacked detectable IEDs. Seizure patterns were not visually discernible in these patients' MEG data, either at the sensor level or in the beamformer‐derived source channels. This precluded source imaging analysis. Seizure patterns were also absent in the simultaneous EEG recordings of Patients ID8 and ID9.

#### 3.5.2 Characteristics and source imaging

**FIGURE 4:**
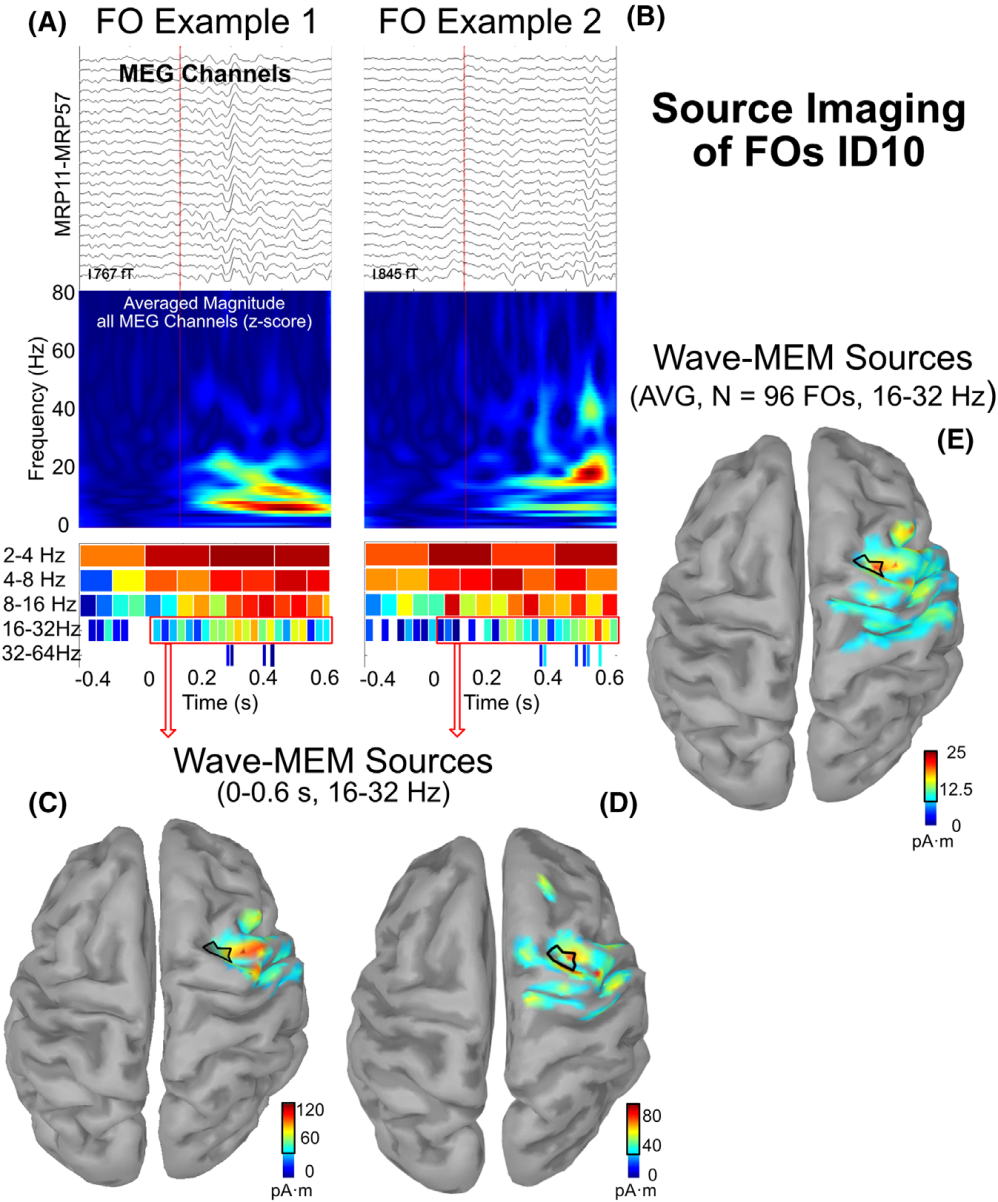
(A, B) Source Imaging of single and averaged fast oscillations (FOs). Two individual FOs in the beta frequency (16–32 Hz) were detected during sleep in Patient ID10. Top: Magnetoencephalography (MEG) sensor traces display multiple oscillatory cycles (red vertical lines mark the onset of the analysis windows). Middle: Time–frequency spectrograms averaged across all MEG channels reveal spectral content concentrated in the beta range. Bottom: Spectral magnitude plots show power across frequency bands over time, with peak activity in the 16–32‐Hz band. (C, D) Wavelet Maximum Entropy on the Mean (wMEM) source imaging for individual FO events (0–.6‐s time window, 16–32‐Hz frequency band) demonstrates focal generators in the right frontal region. (E) Averaged wMEM source from 96 FO events (0–.6‐s window, 16–32‐Hz band) shows robust localization to the right frontal focal cortical dysplasia (FCD) region. This averaged source map represents the spatial distribution of individual wMEM source imaging results from single FO events; the more distributed appearance reflects the spatial variability of where individual FO events localized across the focus. The averaged source map (E) represents the spatial distribution of 96 individual wMEM source localizations; the broader appearance reflects how single FO events varied in their localization across the focus. Black outlines in panels C–E mark the FCD lesion border based on magnetic resonance imaging. Note different color scales (pA·m) between panels to optimize visualization. Averaging multiple events demonstrates the reliability of the wMEM source imaging. The spatial concordance between individual and averaged sources, along with colocalization to the FCD lesion, demonstrates the contributory role of FOs in identifying the seizure onset zone. AVG, average. MRP11‐MRP51, right parietal MEG sensors following the manufacturer's convention.

**FIGURE 5:**
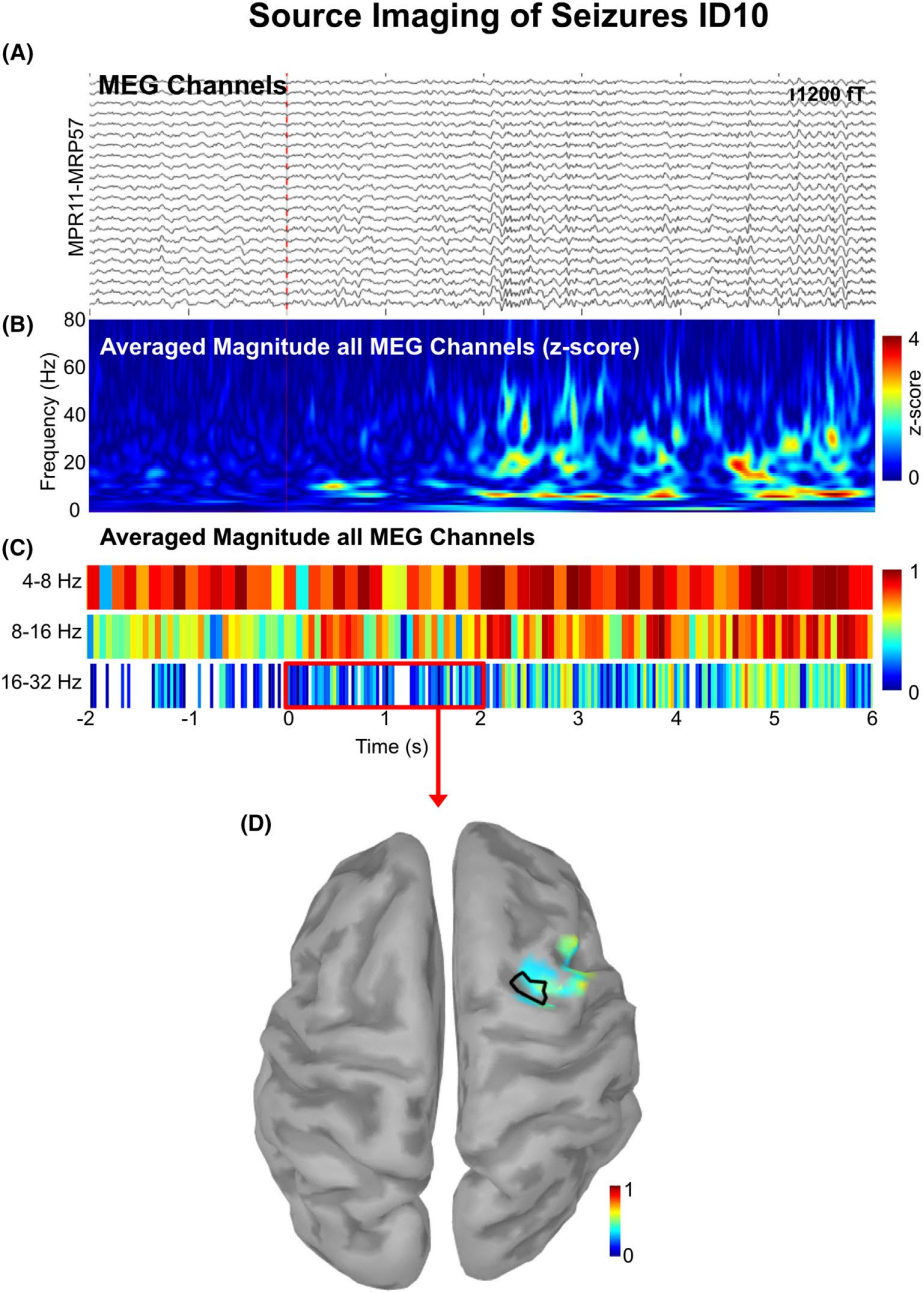
Source imaging of example seizure for Patient ID10. (A) Magnetoencephalography (MEG) sensor traces showing a seizure pattern with onset at t = 0 (red vertical line). (B) Time–frequency spectrogram averaged across all MEG channels reveals spectral evolution. (C) Spectral magnitude plots across frequency bands. Frequency band of interest: 16–32 Hz during seizure; lower frequency activity was judged to be arousal‐related. Red rectangles indicate time windows for wavelet Maximum Entropy on the Mean (wMEM) source analysis. (D) wMEM source localization with higher spatial resolution using an interval of 2 s captures sustained seizure activity localized to the right frontal focal cortical dysplasia (black outline marks lesion border). wMEM enables frequency‐specific source imaging. Its denoising effect allows the extraction and localization of subtle seizure patterns in noisy environments.

FOs occurred almost exclusively during sleep and demonstrated peak activity in beta (14–40 Hz, *n* = 5 patients) and gamma (40–80 Hz, *n* = 2 patients) frequency bands. wMEM source imaging localized FO generators concordant with FCD lesions in five of seven patients (71%), with a median Dmin of 11 ± 15 mm (median ± IQR) in colocalizing patients on a sublobar level (Figure 4). Two patients showed discordant sources, likely influenced by physiological beta activity or residual noise. The averaged source map represents the spatial distribution of individual wMEM source imaging results from single FO events; the more distributed appearance reflects the spatial variability of where individual FO events localized across the focus. Ictal MEG patterns suitable for MSI were identified in four of seven patients (57%) with seizures, with seizure onsets colocalizing with FCD lesions in seven of eight seizures (87.5%, Dmin = 10 ± 17 mm, median ± IQR). Figures 4 and 5 show the localization of FO and seizure using wMEM for example Patient ID10. In this patient, averaged IEDs showed excellent source localization (Dmin = 0 mm; Figure 3), and the localization of FOs and seizures was also concordant with the FCD (Dmin for averaged FOs = 18 mm, seizure = 11 mm). Three patients exhibited clinical seizures without visually identifiable MEG patterns suitable for source analysis; notably, these patients also lacked detectable IEDs. Individual patient results for FO and seizure source localization are detailed in Table S3.

### 3.6 Intracranial EEG and postsurgical outcome

Five patients underwent invasive EEG recordings with sEEG before surgery, and one patient (ID5) underwent intraoperative electrocorticography, confirming the epileptogenicity of the FCD. When comparing lesional ROI with MSI of IEDs in wake and sleep recordings, four of these six patients showed concordant results (defined as sublobar concordance with Euclidean distance ≤ 25 mm to lesion border). The remaining two patients (ID5, ID6) had no IEDs recorded with MEG (Table 1). Regarding fast oscillations, beta FOs in Patient ID1 and gamma FOs in Patient ID5 were concordant with the lesional ground truth (Table S3).

**TABLE 1:** Clinical characteristics of patients.

ID	Age, years	Age at onset, years	ASM	Seizure types	FCD localization (SRE LTM)	VEEG, IEDs/SOZ	invEEG	Surgery	MRI/histopathology	Outcome	Follow‐up
1	29	2	LTG, LEV	ICS, BTCS	F R (+)	R F C/R F C	sEEG: IZ/SOZ conc.	Yes	mMCD	IA	18
2	19	17	LTG, BRV	ICS, BTCS	P L (+)	R F C/R F C	sEEG: IZ/SOZ conc.	Yes	Type II	IB	42
3	54	28	LTG, BRV	ICS	Ci L	L T F/L F T		Yes	Transmantle FCD/no diagnosis, too fractionated	IA	45
4	19	51	LEV, OXC	PCS, ICS	F R (+)	R F C/R F C			Transmantle FCD		
5	34	12	BRV	ICS, BTCS	F R (+)	R F T/R F T	intraop. ECoG IZ/SOZ conc.	Yes	Type II	IA	12
6	36	15	LTG, CNB	PCS, BTCS	F L	L F C/no seizure pattern	sEEG: IZ/SOZ conc.	Yes	Type II	IB	12
7	26	15	LTG	PCS, ICS, BTCS	F R	R F C/R F C	sEEG: IZ/SOZ conc.	Yes	mMCD	IB	6
8	42	7	VPA, LEV, LTG	PCS, BTCS	F R (+)	R F/R F		Yes	Type II	IA	12
9	35	14	VPA, LEV	PCS, BTCS	F R	No IEDs/no seizure pattern			Transmantle FCD		
10	26	18	LCM, LTG, LEV	PCS, BTCS	F R	R C (no VEEG)			Transmantle FCD		
11	45	16	LCM, LEV	ICS, BTCS	T O R, P R (+)	R T O/R T O		Yes	Type II	IA	12
12	61	16	BRV, LCM	ICS	F R (+)	R F/R F C			Suspected FCD based on MRI criteria		
13	32	2	PB, PHE, ZNS, CNB, LEV	PCS, ICS	F L (+)	L C/L C			Transmantle FCD		
14	67	16	BRV, LTG, CNB	PCS, ICS, BTCS	F R	R F T/R F T	sEEG: IZ/SOZ conc.	Yes	Type II	IA	7
15	37	4	LEV, CNB	PCS, ICS, BTCS	F L (+)	L F C/L F C			Transmantle FCD		
16	29	7	LCM	PCS, BTCS	F C L	None (no VEEG)			Transmantle FCD		
17	53	10	BRV, CNB	ICS, BTCS	F L (+)	None/L F C		Yes	Transmantle FCD/gliosis (too fragmented)		
18	25	6	OXC, PER, CNB	PCS, ICS	R P (+)	Bi‐F C T/R P O			Transmantle FCD		
19	28	5	BRV, LTG	PCS, ICS, BTCS	F R	R F C/R F C			Transmantle FCD		

*Note*: "Age" refers to age at time of study. "Age at onset" refers to age at epilepsy onset. "Outcome" refers to Engel classification (IA, completely seizure‐free; IB, nondisabling simple partial seizures only). "Follow‐up" refers to duration of postoperative follow‐up (months). "Type II" indicates FCD type II (subclassified as IIa or IIb). "Transmantle FCD" indicates FCD with transmantle sign.

Abbreviations: ASM, antiseizure medication; BRV, brivaracetam; BTCS, focal to bilateral tonic–clonic seizures; C, central; Ci, cingulate; CNB, cenobamate; conc., concordance with epileptogenic lesion based on MRI criteria; ECoG, electrocorticography; EEG, electroencephalography; F, frontal; FCD, focal cortical dysplasia; ICS, (focal) impaired consciousness seizures; ID, patient identifier; IED, interictal epileptiform discharge; intraop., intraoperative; invEEG, invasive EEG; IZ, irritative zone; L, left; LCM, lacosamide; LEV, levetiracetam; LTG, lamotrigine; mMCD, mild malformation of cortical development; MRI, magnetic resonance imaging; O, occipital; OXC, oxcarbazepine; P, parietal; PB, phenobarbital; PCS, (focal) persevered consciousness seizures; PER, perampanel; PHE, phenytoin; R, right; sEEG, stereo‐EEG; SOZ, seizure onset zone; SRE LTM, sleep‐related epilepsy with more than 2/3 of seizures from sleep during long‐term VEEG; T, temporal; VEEG, video‐EEG monitoring; VPA, valproic acid; ZNS, zonisamide.

Nine patients underwent epilepsy surgery, with postsurgical outcome data available for eight patients (minimum follow‐up > 6 months). All eight achieved Engel I outcome, with mean follow‐up of 18 ± 15 months (Table 1). Among the nine surgical patients, six had MSI of IEDs concordant with lesional ground truth, whereas three patients lacked IEDs in MEG recordings (ID3, ID6, ID7).

Fewer patients with FOs or seizures recorded by MEG proceeded to surgery. Among patients with FOs, three (ID1, ID5, ID11) showed concordance with the lesional area. For seizure patterns, two patients (ID11, ID14) demonstrated concordance with the lesional area. Patient ID8 experienced a seizure but showed no seizure pattern in MEG and lacked IEDs in both MEG and EEG recordings. Among patients who proceeded to surgery, no discordant results (sublobar mismatch or Euclidean distance > 25 mm to lesion border) for FOs or seizures occurred. Histopathology results are presented in Table 1.

## 4 DISCUSSION

### 4.1 Main findings

This prospective study demonstrates that incorporating daytime sleep into MEG/EEG protocols adds relevant information to the epilepsy evaluation of FCD patients. Sleep recordings captured seizures in seven of 19 patients (37%) and FOs in seven of 12 patients with IEDs (58%), biomarkers with high specificity for the epileptogenic zone that were rare or absent during wake recordings. Both seizure rates and FO rates increased significantly during sleep (both *p* < .05), whereas IED rates showed nonsignificant trends. Quantitative metrics of IED‐based MSI were stable between wake and sleep recordings. The wMEM inverse method enabled frequency‐specific source imaging of FOs and seizures, with sublobar colocalization to FCD lesions in five of seven patients with FOs and four of seven patients with seizures.

Our findings provide quantitative evidence that standard wake‐only MEG protocols miss clinically critical information in FCD patients.^50^ The protocol modifications required are minimal—extending recording duration to approximately 90 min to capture natural daytime sleep—yet yield substantial diagnostic gains. Although we studied FCD patients as a model system with anatomical ground truth for validation, the sleep enhancement of seizure and FO detection has direct implications for nonlesional focal epilepsy, where precise noninvasive localization is most critical for surgical planning.

### 4.2 IED source imaging accuracy and comparison with prior studies

MSI maxima of IEDs demonstrated good accuracy in both vigilance states, with median Euclidean distances of 12.74 mm during wake and 8.34 mm during sleep to FCD lesions. These spatial accuracy metrics are consistent with previous MSI studies of IEDs in focal epilepsy.^39^, ^45^, ^51^ The robust localization across vigilance states indicates that sleep‐based MEG recordings provide reliable epileptogenic zone identification comparable to wake recordings.

### 4.3 Source imaging of FOs and seizures

wMEM enabled clinically relevant MSI of seizures and FOs with frequency‐specific and high temporal resolution, exceeding conventional methods that typically require averaging of 4–5 s of data.^52^ This enhanced temporal precision proved particularly valuable for FOs, which occurred more frequently than seizures and thus potentially allowed for more robust localization through averaging multiple events.^43^, ^44^

Source imaging success varied across patients, with the best results in those showing well‐localized IEDs, suggesting that favorable MEG signal characteristics predict localization quality across all types of interictal and ictal activity. Importantly, when multiple biomarkers were successfully localized, spatial concordance between IED sources, FO generators, and SOZs provided independent converging evidence for the epileptogenic zone. This multibiomarker validation represents a key clinical advantage; concordant localization of IEDs, FOs, and seizures with lesional ROIs assessed through Dmin and sublobar colocalization increases confidence in noninvasive findings and may reduce the need for invasive monitoring in select patients.

### 4.4 Comparison with previous invasive EEG and MEG studies

Sleep recordings revealed both increased FO rates and seizure occurrence in FCD patients, providing converging evidence for sleep‐related epileptogenicity. Our MEG recordings detected FOs in both beta and gamma frequency bands during sleep,^26^ consistent with invasive EEG observations in FCD patients.^11^, ^12^ MEG's superior sensitivity for superficial and tangential cortical sources compared to scalp EEG,^53^ combined with reference‐free sensors and silent operation that facilitate sleep recordings, enabled robust noninvasive detection of these biomarkers. Although MEG FO rates are lower than in scalp EEG, MEG FOs in the ripple band demonstrate higher specificity and localization accuracy for the epileptogenic zone.^26^, ^27^ The sleep‐related increase in FOs may represent a pathophysiological correlate underlying increased seizure risk during sleep.^54^ We confirmed increased seizure rates during sleep in our cohort,^18^, ^19^ with 11 of 19 patients (58%) showing documented sleep‐related epilepsy. Increased FO rates during sleep were observed in both frontal and extrafrontal lobe epilepsy (ID11, ID18; Table S3), supporting broad applicability of sleep recording protocols. The parallel increases in both FO rates and seizure occurrence during sleep suggest a potential mechanistic link, although establishing causality would require comparison with appropriate control groups. Sleep‐dependent FOs appear to be a shared feature across epilepsy types, also observed, for example, in childhood epilepsy with centrotemporal spikes^55^ and infantile spasms on scalp EEG.^56^

Our cohort achieved uniform surgical success (8/8 patients Engel I), preventing outcome prediction; future prospective studies with variable postsurgical outcomes are needed to determine whether spatial concordance of IEDs, FOs, and seizures predicts favorable outcome in MEG, a hypothesis supported by high‐density EEG studies demonstrating that spatial association of FOs with IEDs predicts favorable surgical outcome.^57^

### 4.5 Differences in IED and slow wave amplitudes and changes in source extent

Analysis of IED and slow wave amplitudes revealed no systematic changes between sleep and wake states. The primary factor influencing SD and Dmin was the number of averaged IEDs rather than vigilance state, with a trend for negative correlations between IED count and both SD and Dmin. These findings suggest that noise levels in noninvasive MEG recordings obscure spatial extent changes reported in invasive studies,^58^ consistent with overnight high‐density EEG results.^46^ Systematically demonstrating spatial extent differences from noninvasive data remains challenging.

### 4.6 MRI ROI‐based review of MEG signal

Our analysis benefited from applying an LCMV beamformer to identify IEDs and FOs at the source level, an approach systematically validated for both signal types.^27^, ^33^ Visual review was restricted to predefined lesional and contralateral control ROIs rather than all ~1284 cortical vertices for practical feasibility and to leverage the beamformer's optimal spatial filtering and denoising properties.^34^ The contralateral homologous ROI served as an anatomically matched control. Source‐level data provided enhanced visibility of IEDs and FOs compared to sensor‐level detection. Retaining both sensor‐level MEG/EEG data and source‐level analysis offers complementary advantages: focal precision at the source level while preserving whole‐brain spatial information. This methodology may prove valuable when combined with MRI morphometry, which often generates false‐positive clusters. Future quantitative integration of MSI with MRI morphometry could validate MSI as a positive predictor for favorable postsurgical outcomes.^9^, ^13^

### 4.7 Clinical implications

Incorporating daytime sleep into MEG protocols substantially enhances biomarker detection with minimal additional effort. Extended 90‐min recordings proved feasible and safe, with no adverse events despite capturing seizures. This protocol balances diagnostic yield and resource requirements compared to overnight recordings.

Beyond FCD evaluation, these results have implications for nonlesional focal epilepsy, where noninvasive multibiomarker concordance (IEDs, FOs, seizures) could guide sEEG implantation planning. Figure S6 illustrates this application in a patient with mMCD, where MEG source imaging of sleep‐enhanced beta‐band FOs showed spatial concordance with the SOZ subsequently identified by invasive sEEG. Such concordance supports prospective studies evaluating whether MEG‐guided strategies enable more targeted electrode placement or, when multiple biomarkers show robust spatial concordance, increase confidence for proceeding to resection without invasive monitoring in selected patients.^59^ The sleep‐enhanced detection of seizures and FOs may be beneficial to guide placement of intracranial EEG electrodes in patients with nonlesional focal epilepsy, patients with discordant MRI and video‐EEG monitoring, and patients with subtle or MRI‐negative lesions where MSI based on IEDs was already of advantage.^60^ MEG demonstrates distinct advantages over EEG for detecting tangential sources in opercular and peri‐insular regions,^8^, ^59^, ^61^ locations represented in frontal‐predominant FCD localizations.^6^

### 4.8 Limitations

This study focused on FCD patients to enable validation against anatomical ground truth; applicability to other epilepsy etiologies requires confirmation. Visual identification of FOs requires expert review and may underestimate true event rates compared to automated detection. Daytime sleep recordings captured primarily N1–N2 sleep stages; overnight recordings would enable more detailed sleep‐stage analysis but require substantially greater resources. The balance between diagnostic yield and practical feasibility suggests daytime protocols offer optimal cost‐effectiveness. The small number of patients with FOs (*n* = 3) and seizures (*n* = 2) who proceeded to surgery precluded assessment of these biomarkers' predictive value for postsurgical outcome, despite their demonstrated concordance with lesional tissue. Larger prospective studies are needed to confirm these findings, particularly in nonlesional focal epilepsy patients, where precise noninvasive localization is most critical for surgical planning.

## 5 CONCLUSIONS

Extended daytime MEG/EEG recordings incorporating sleep optimize presurgical evaluation by capturing FOs and seizures that standard wake recordings frequently miss. Our protocol demonstrated high clinical feasibility, with sleep achieved in N1 (89%) and N2 (73%) of patients and substantial diagnostic gains during sleep states. Seizures provide the gold standard SOZ localization, and FOs offer additional independent localization information complementing conventional IED analysis. MSI of IEDs yields consistent localization accuracy regardless of vigilance state. The wMEM method enables clinically relevant source imaging of FOs and seizure patterns, with multibiomarker spatial concordance providing validation of the epileptogenic zone.

The enhanced detection and localization of clinically relevant biomarkers support the adoption of sleep‐inclusive protocols as standard practice in presurgical MEG evaluation. This approach optimizes MEG's contribution within multimodal workup by maximizing information extraction from each study. Through more precise noninvasive localization, sleep protocols may improve invasive monitoring strategies by enabling more targeted electrode placement or, when multiple biomarkers show concordant localization, increasing confidence for proceeding without invasive evaluation in selected patients.

## FUNDING INFORMATION

This project was funded by the German Research Foundation (project ID: 468174690; Y.L.H. [LI1904/2–1] and M.H. [HE6844/3–1]).

## CONFLICT OF INTEREST STATEMENT

A.S.‐B. has received research support from Bial, Precisis, and UNEEG, and personal honoraria for lectures or advice from Angelini Pharma, Jazz/GW Pharmaceuticals, Precisis, UCB, and UNEEG. H.U. has received honoraria for lectures from Biogen, Eisai, Mbits, Lilly, and Bayer, is supported by the German Federal Ministry of Education and Research, and is coeditor of *Clinical Neuroradiology*. M.H. has received support for conference participation from Jazz/GW Pharmaceuticals and Precisis, and speaker's honoraria for lectures from Eisai and Arkana. H.L. has be received research support from Bial, Boehringer Ingelheim, and Lario Therapeutics; he is a consultant for Lario and Praxis Precision Medicine, and a former consultant for Angelini, Bial, Eisai, and UCB. T.D. is a consultant for Medtronic, and has received travel and educational grants from Balt, Stryker, and Medtronic. None of the other authors has any conflict of interest to disclose. We confirm that we have read the Journal's position on issues involved in ethical publication and affirm that this report is consistent with those guidelines.

## Supporting information


Data S1.

## Data Availability

The data that support the findings of this study are available on request from the corresponding author. The data are not publicly available due to privacy or ethical restrictions.
