# Supplementary material for: Sleep magnetoencephalography enhances detection and source imaging of seizures and fast oscillations in focal cortical dysplasia
Source: Epilepsia. 2026 Mar 10;67(6):2992–3008. doi: 10.1002/epi.70191 (PMC13285261; doi:10.1002/epi.70191)
Supplement: Supplementary file 1 — Data S1. [file EPI-67-2992-s001.pdf]

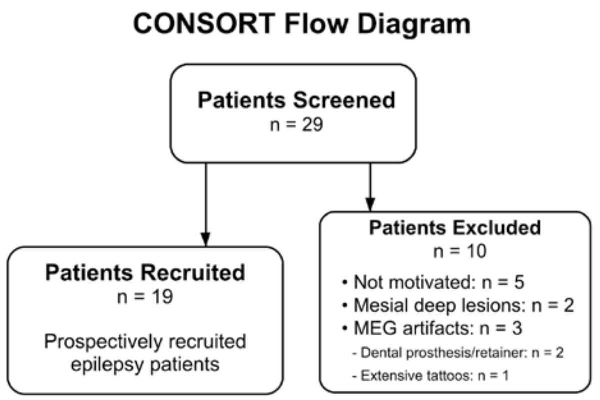

Suppl. Fig. 1: CONSORT chart for prospective patient recruitment.

Stage	Mean	Median	SD	N
**AWA**	32.9	33.0	20.1	19
**N1**	20.6	21.5	11.9	19
**N2**	28.1	24.5	18.7	14
**N3**	5.0	5.0	0.0	1
**Total**	78.9	88.5	18.5	19

Suppl. Table 1. Sleep stage distribution during MEG recordings. AWA: awake state; N1: non-REM stage 1 sleep; N2: non-REM stage 2 sleep; N3: non-REM stage 3 sleep; SD: standard deviation; N: number of patients. Values represent duration in minutes.

A

Parameter	Wake State Median (IQR)	Sleep State Median (IQR)	p-value	Effect Size (r)
**Source Dispersion (SD)**	21.55 (22.96)	17.55 (15.11)	0.735	0.11
**Dmin (Euclidean distance)**	12.74 (22.74)	8.34 (27.58)	0.892	0.04
**AUC (localization accuracy)**	0.68 (0.21)	0.72 (0.14)	0.441	0.25
**IED Amp. (fT)**	615.50 (205.50)	604.50 (382.50)	0.678	0.13
**Slow Wave Amp. (fT)**	287.50 (182.00)	401.00 (257.00)	0.234	0.38

B

**Parameters**		**SD**		**Dmin (mm)**		**AUC**		**IED Amp. (fT)**		**Slow Wave Amp. (fT)**	
**ID**	**IED Count**	**Wake**	**Sleep**	**Wake**	**Sleep**	**Wake**	**Sleep**	**Wake**	**Sleep**	**Wake**	**Sleep**
**P01***	8	19.81	11.8	7.15	0	0.67	0.69	466	371	287	421
**P02***	5	45.29	27.36	25.04	30.68	0.85	0.77	516	559	481	481
**P04**	11	42.79	43.92	33.8	34.27	0.49	0.3	641	715	1073	854
**P05**	682	12.75	11.9	0	0	0.75	0.76	571	579	141	139
**P08***	7	31.1	25.53	24.48	24.48	0.65	0.62	620	363	377	271
**P10**	74	9.27	11.36	0	0	0.81	0.9	419	478	277	489
**P11***	22	11.76	12.92	4.04	8.34	0.77	0.72	712	1061	255	381
**P12**	14	69.06	39.69	25.96	45.75	0.36	0.71	266	391	239	229
**P13**	8	23.3	17.23	21.95	0	0.7	0.74	681	806	288	484
**P14***	56	27.74	27.67	11.07	19.1	0.54	0.46	922	828	344	230
**P15**	55	16.32	14.31	0	0	0.6	0.62	611	630	213	173
**P18**	31	15.23	17.87	14.41	8.35	0.8	0.83	1130	1228.5	688	656.5

Suppl. Table 2: Quantitative comparison between wake and sleep source imaging findings. Table A presents summary statistics showing statistical comparison of source imaging parameters between wake and sleep states across all patients (N=12 patients with interictal epileptiform discharges), with data presented as median (interquartile range (IQR)) and p-values derived from Wilcoxon signed-rank tests with effect size (r) calculated as $Z/\sqrt{N}$. Table B shows individual patient data with measurements for each patient (P01-P12) displaying wake and sleep state values for all parameters. Parameters include SD (Source Dispersion), representing the spatial dispersion of the source imaging measured in millimeters; Dmin (Euclidean distance), indicating the minimum Euclidean distance from source to lesion in millimeters; AUC (Area Under the Receiver Operating Characteristic Curve), serving as a localization accuracy metric ranging from 0 to 1 where higher values indicate better localization; IED (Interictal Epileptiform Discharges) amplitude measured in femtotesla (fT); Slow Wave Amp., representing the amplitude of slow wave activity measured in femtotesla (fT); and IED Count, indicating the number of IEDs analyzed per patient. *epilepsy surgery

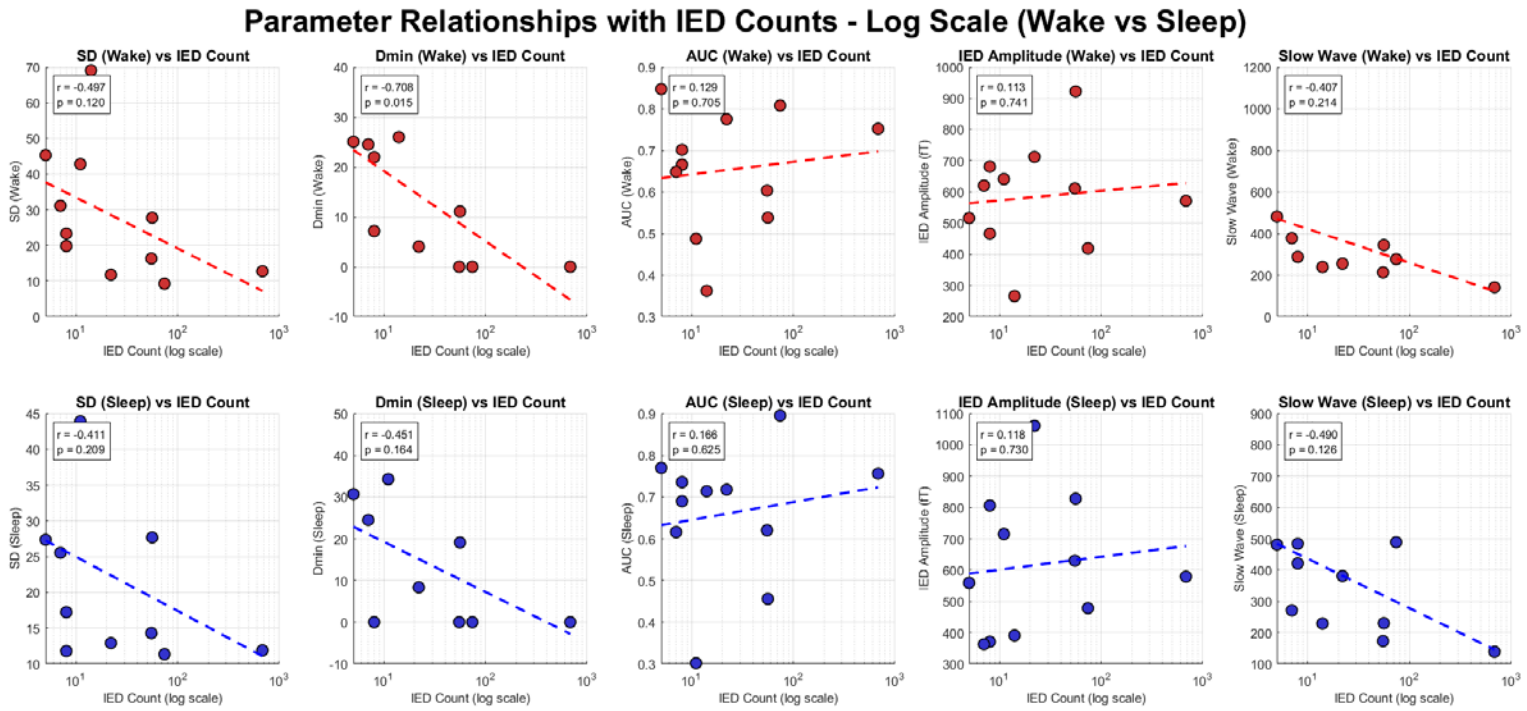

Suppl. Fig. 2: Relationship between IED count and localization metrics, showing improved precision with higher IED rates for both wake and sleep recordings. AUC: Area-under-the-receiver-operating-characteristics-curve, Dmin: Euclidean distance, IED: interictal epileptiform discharges, SD: spatial dispersion.

			Fast oscillations				Seizures			
**ID**	Lesion	IEDs	Dmin	Band	N	Conc.	Dmin	Pattern/ Band	N	Conc.
**ID1***	FOR	+	7	Beta	298	Yes	-	-	-	-
**ID5***	FPR	+	0	Gamma	130	Yes	-	-	-	-
**ID8***	-	-	-	-	-	-	-	-	1	NA
**ID9**	-	-	-	-	-	-	-	-	3	NA
**ID10**	FLSR	+	18	Beta	98	Yes	11/17/ 53§/18	Rhythmic/ Beta	3	Yes (3/4)
**ID11***	TLPR	+	22	Beta 2	18	Yes	1	Rhythmic/ Beta 2	1	Yes
**ID12**	FPR	+	117†	Beta	83	No	-	-	-	-
**ID13**	FLSR	+	-	-	-	-	0/0	Rhythmic/ Gamma	7	Yes (2/7)‡
**ID14***	FOR	+	-	-	-	-	24	Rhythmic/ Beta	1	Yes
**ID15**	FOR	+	91§	Beta	67	No	-	-	-	-
**ID18**	POMR	+	11	Gamma	130	Yes	-	-	-	-
**ID19**	FLSR	-	-	-	-	-	-	-	1	NA‖

Suppl. Table 3. Fast Oscillation and Seizure Source Imaging Results Using wavelet-MEM (wMEM). Band: Frequency band, Dmin, Euclidean distance in mm from source maximum to FCD lesion border; FOR, fronto-orbital right; FPR, fronto-polar right; FLSR, fronto-lateral superior right; TLPR, temporo-lateral posterior right; POMR, parieto-occipital mesial right; IEDs, interictal epileptiform discharges; FO, fast oscillations; Sz, seizures; N, number of events analyzed; NA, no seizure pattern could be detected (these patients also lacked detectable IEDs). Beta = 16-32 Hz; Beta 2 = 25-50 Hz (ID11); Gamma = 40-80 Hz (ID5, ID18) or 50-100 Hz (P13). Seizure onsets showed rhythmic beta-gamma patterns consistent with FCD phenotype (Tassi et al. 2012, Chassoux et al. 2012). Frequency bands for wave-MEM source imaging were individually adapted based on spectrograms of each patient's seizures and fast oscillations. Spatial concordance (Conc.) was evaluated at the sublobar level based on Heers et al. Brain Topogr. 2016. §False localization. †Residual noise influenced source accuracy. §Strong physiological beta activity influenced source accuracy. ‡Five seizures were excluded due to artifacts. ‖ Seizure pattern in EEG but not in simultaneous MEG data due to strong movement artifacts in MEG.* epilepsy surgery

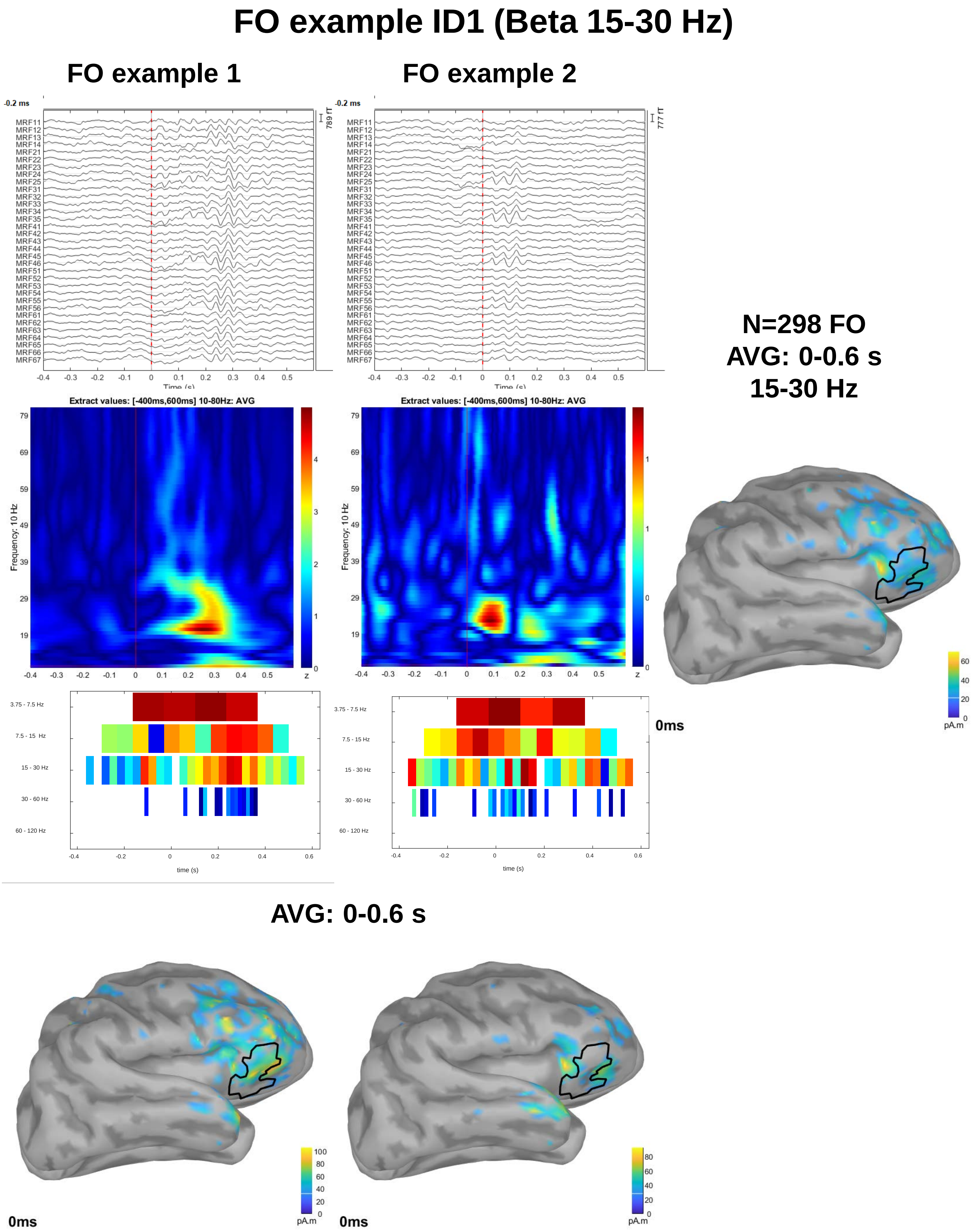

Suppl. Fig. 3: Wavelet-MEM source imaging of gamma frequency fast oscillations (15-30 Hz) in Patient ID1. Two individual FO events (top) and averaged source from 298 FOs (bottom) demonstrate focal localization to right fronto-orbital FCD (black) outline.

FO example ID18 (Gamma 40-80 Hz)

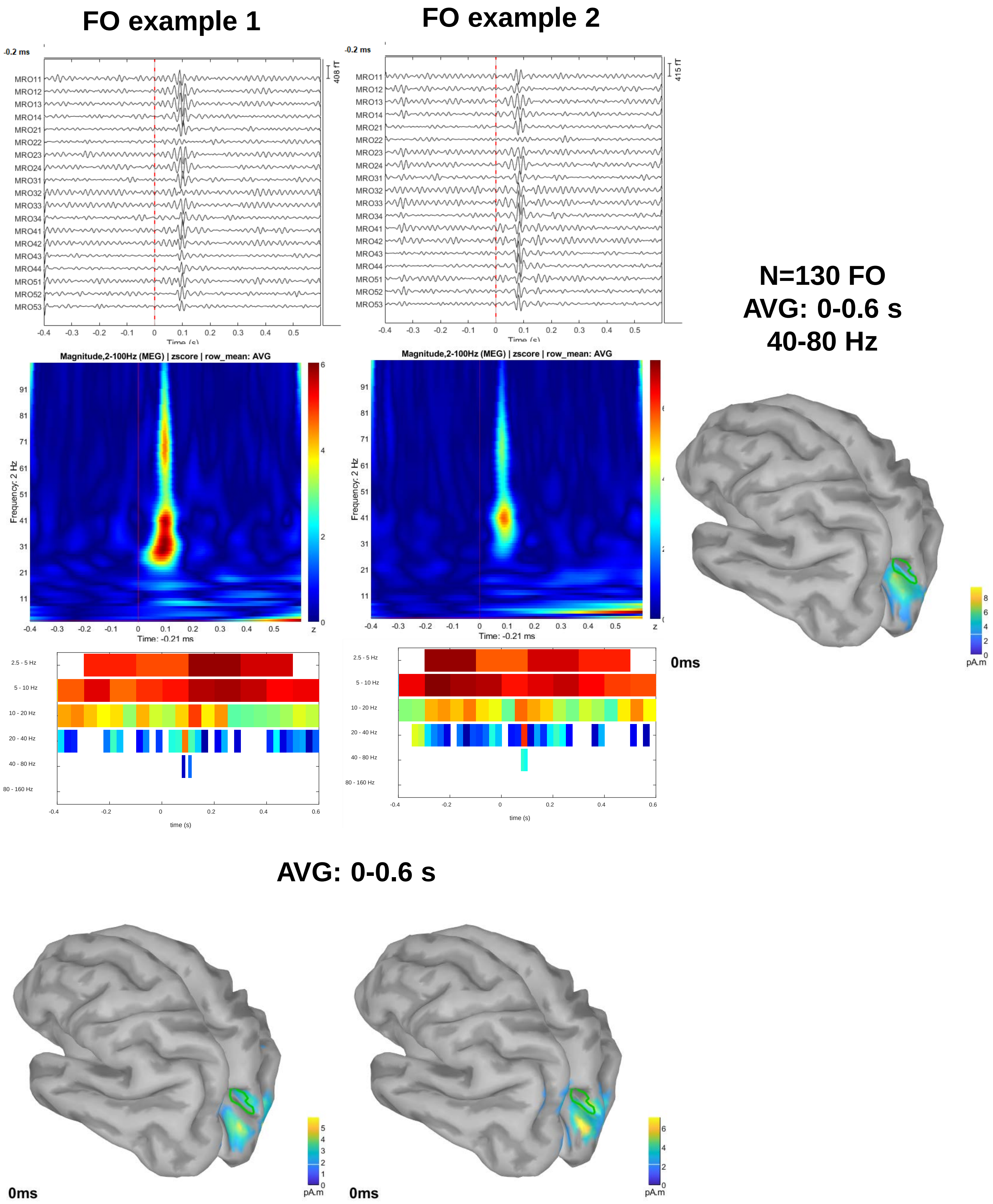

Suppl. Fig. 4: Wavelet-MEM source imaging of gamma frequency fast oscillations (40-80 Hz) in Patient ID19. Two individual FO events (top) and averaged source from 130 FOs (bottom) demonstrate focal localization to right fronto-polar FCD (green) outline.

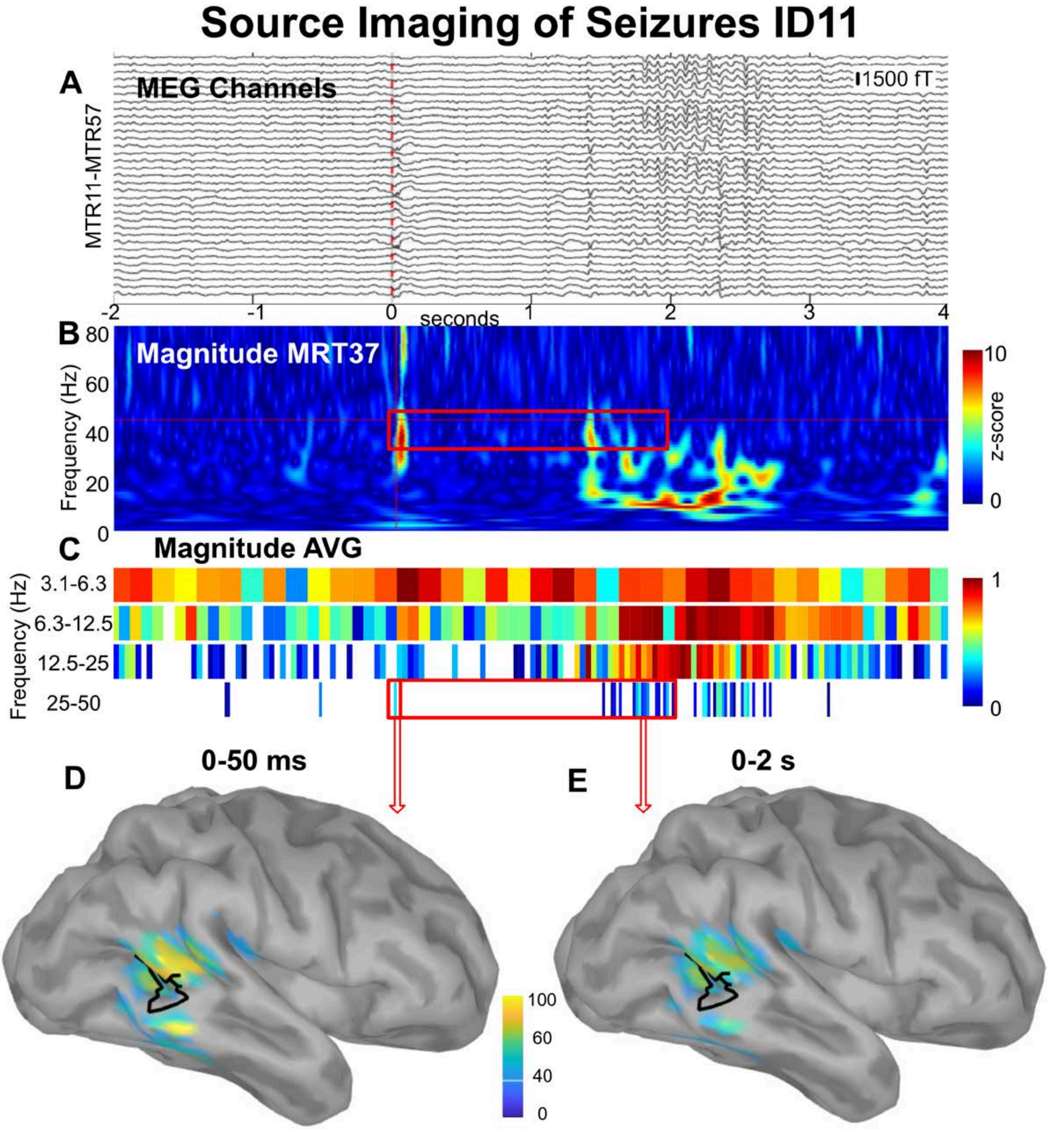

Suppl. Fig. 5: Seizure source imaging (Patient ID11). **(A)** Seizure pattern visible in MEG traces across multiple channels (onset marked by red vertical line at t=0). **(B)** Time-frequency analysis shows spectral evolution during the seizure. **(C)** Discrete wavelet analysis identifies the most relevant time-frequency windows for source localization. **(D, E)** Wavelet-MEM source imaging localizes seizure generator to right temporo-occipital FCD region (black outline). **(D)** Source localization using the 0-50 ms interval in the 25-50 Hz frequency band highlighting the high temporal resolution of the Wave-MEM method. **(E)** Source localization using the 0-2 s interval in the 16-32 Hz frequency band.

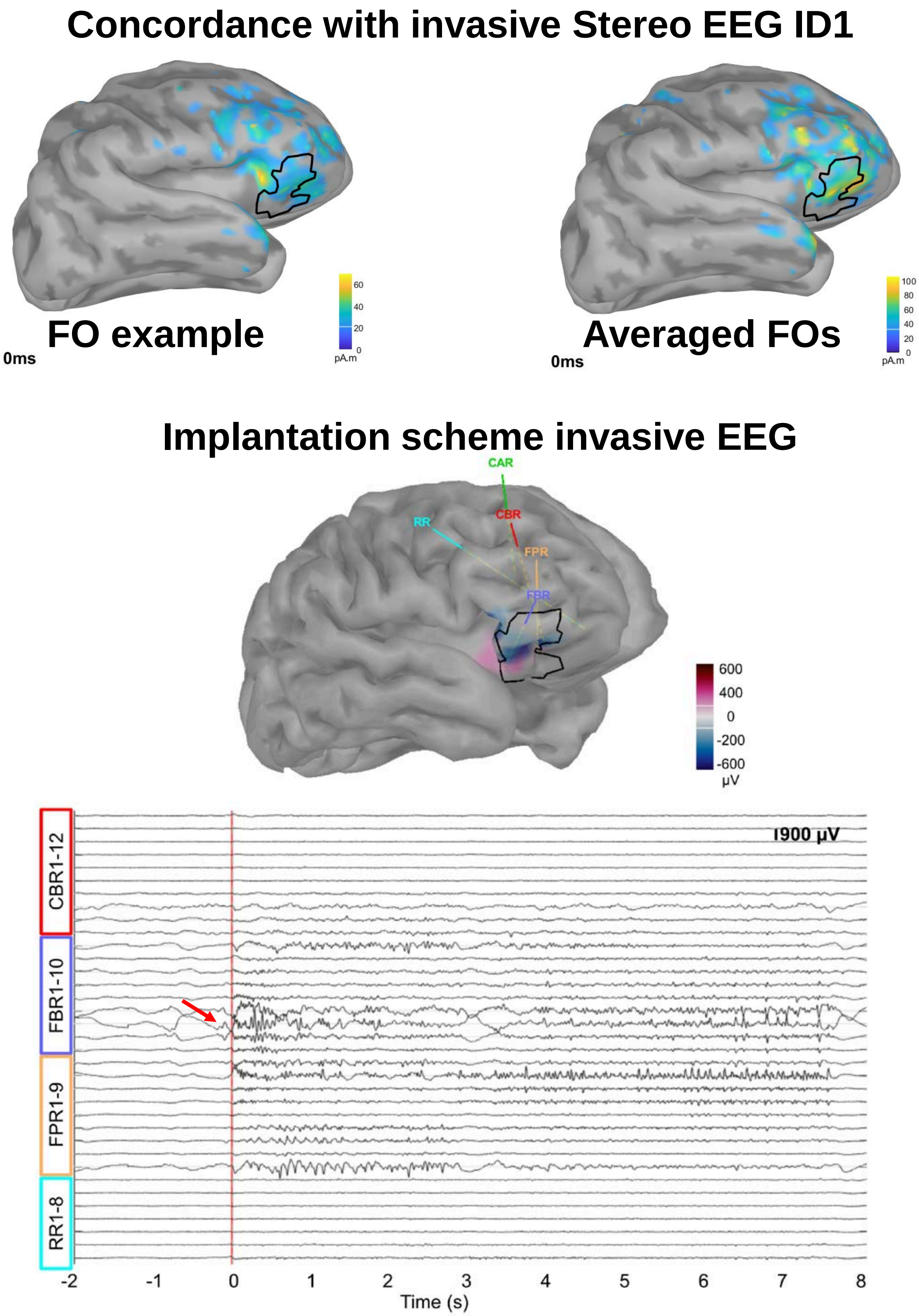

Suppl. Fig. 6: Concordance of invasive stereo-EEG activity with magnetic source imaging of fast oscillations (FOs) in right frontal lobe epilepsy (P1) Upper panel: Wave-MEM source imaging of beta-frequency (15–20 Hz) FOs at seizure onset. Left, single FO example; right, averaged FO activity (N=298). Black lines indicate lesion borders. Heatmaps show source power. Center panel: Stereo-EEG implantation scheme with 6 intracerebral depth electrodes. FBR electrode at lesion center; contacts FBR6–8 within seizure onset zone (red arrow). Heatmap overlay shows stereo-EEG amplitude at seizure onset (t=0) projected on cortical surface. Lower panel: Bipolar raw electrocorticographic traces from 4 representative depth electrodes at seizure onset (red vertical line at t=0 s).
